# Post-Compulsory Education in Teenagers and Young Adults Treated for Brain Tumors in Childhood: A Swedish Nationwide Registry-Based Study

**DOI:** 10.3390/cancers15010255

**Published:** 2022-12-30

**Authors:** Malin Lönnerblad, Maria Åberg, Klas Blomgren, Eva Berglund

**Affiliations:** 1 Department of Women’s and Children’s Health, Uppsala University, Akademiska Sjukhuset, 75185 Uppsala, Sweden; 2 Department of Women’s and Children’s Health, Karolinska Institutet, 171 64 Stockholm, Sweden; 3 Department of Special Education, Stockholm University, 10691 Stockholm, Sweden; 4 School of Public Health and Community Medicine/Primary Health Care, Institute of Medicine, University of Gothenburg, Sahlgrenska Academy, 40530 Gothenburg, Sweden; 5 Regionhälsan, Region Västra Götaland, 40583 Gothenburg, Sweden; 6 Pediatric Oncology, Karolinska University Hospital, 17164 Stockholm, Sweden

**Keywords:** pediatric brain tumors, embryonal tumors, low-grade astrocytomas, optic pathway gliomas, craniopharyngiomas, neuronal and mixed neuronal–glial tumors, post-compulsory education, registry-based study

## Abstract

**Simple Summary:**

Individuals treated for brain tumors in childhood are at high risk of cognitive and other late complications. The aim of this nationwide registry study was to explore further education following the nine years of compulsory school in Sweden in teenagers and young adults treated for childhood brain tumors. Individuals treated for embryonal tumors, low grade astrocytomas, optic pathway gliomas, craniopharyngiomas, and neuronal and mixed neuronal–glial tumors, were analyzed separately. All individuals treated for brain tumors were compared to about five times as many matched controls without cancer diagnoses or treatments. Our results demonstrate significant differences between cases and controls regarding attendance in high school, folk high school, and university. Individuals treated for embryonal tumors or optic pathway gliomas attended post-compulsory education less frequently than other analyzed diagnoses. There was a positive correlation between parental education levels and attendance in high school and university for both cases and controls.

**Abstract:**

The risk of late complications after a brain tumor in childhood is high. Both the tumor itself and the treatments give rise to sequelae that affect daily life activities. In this registry study, we explored post-compulsory education, i.e., further education following the nine compulsory years in school, in 452 cases born 1988–1996 and diagnosed with a brain tumor before their fifteenth birthday. They were compared with 2188 individual controls who were not treated for cancer. Significantly fewer teenagers and young adults treated for brain tumors in childhood attended high school or university compared with controls, especially individuals treated for embryonal tumors or optic pathway gliomas. A significantly larger proportion of subjects treated for embryonal tumors and craniopharyngiomas attended folk high schools, a type of post-compulsory school with a more accessible learning environment. For both cases and controls, we observed a positive correlation between parental education levels and attendance in high school and university. In our previous studies we have shown that children treated for brain tumors, as a group, tend to perform worse during their last year of compulsory school compared with their peers, and the current study confirms that these differences remain over time.

## 1. Introduction

During the period 1984–2005, the brain tumor incidence for children under the age of 15 was 4.4 out of 100,000 in Sweden, with an average survival rate of about 80%, although with large variation depending on tumor type [1,2]. Individuals treated for brain tumors in childhood, hereafter termed pediatric brain tumor survivors (PBTS), are at high risk of cognitive late complications [3,4,5] after their tumor and treatment. They may suffer from fatigue, reduced processing speed and attention, as well as impaired working memory [4,6,7,8,9,10,11,12,13]. Many children are also at risk for hearing loss [14]. The risk of more severe late complications are correlated to younger age at diagnosis [4,6,12,13] and multiple studies report that girls seem to be more severely affected than boys [4,15,16,17]. Children treated for high-grade tumors, for example embryonal tumors, generally have a high risk of late complications [18,19] which can lead to academic difficulties [20,21]. However, there is an increasing number of studies showing a high risk of late complications following treatment also for different low-grade tumors [22,23,24,25]. For example, many children diagnosed with optic pathways gliomas experience visual impairment to some degree [26,27]. In about 30% [28] of the cases, optic pathways gliomas are associated with neurofibromatosis type 1, a genetic disorder that often also leads to further late complications such as inattention, visuospatial deficits, and mild intellectual disability which may affect academic performance [27,29,30]. Children treated for craniopharyngiomas are known to be at risk for behavioral changes, fatigue, and cognitive deficits that also may affect academic performance [31,32,33].Children treated for low-grade astrocytomas and neuronal and mixed neuronal–glial tumors have also been shown to be at risk for late complications [25,34]. Problems resulting from late complications often increase over time [4,35,36] and there is a high risk of difficulties during compulsory school [23,37,38,39]. Previous studies have shown that PBTS less frequently attended post-compulsory education [37,40], i.e., further education after the nine years of compulsory school, and were employed to a lesser extent compared to controls without childhood brain tumor diagnoses [41,42].

The aim of this study was to explore post-compulsory educational attendance for teenagers and young adults treated for brain tumors in childhood in Sweden. Differences in education categorized by sex, age at diagnosis, parental education, and the different tumor types (embryonal tumors, low-grade astrocytomas, optic pathway gliomas, craniopharyngiomas, and neuronal and mixed neuronal glial tumors) were also investigated. There is only limited information in the literature on to what extent these groups attend different types of post-compulsory education, such as high school, municipal adult education, university, as well as folk high school, a type of post-compulsory school with a learning environment known to be more adapted to individual needs [43,44]. This is important, since the risk of unemployment in Sweden is particularly high for young adults without post-compulsory education and young adults with disabilities [45].

## 2. Materials and Methods

### 2.1. Participants and Data Collection

Individuals from Sweden born 1988–1996 and diagnosed with a brain tumor before their fifteenth birthday were included in this study. In three previous studies [23,46,47], we investigated both the final grades from school year nine and the national test grades that preceded the final grades for these children. Here, the same cohort was included, excluding PBTS and controls who were no longer alive in 2016 (PBTS, *n* = 23; 4.84%; controls *n* = 9; 0.4%), yielding 452 PBTS and 2188 unique controls without any history of cancer (Table 1). For more information about inclusion and exclusion, see Lönnerblad et al., 2021. Data about PBTS were obtained from the Swedish Childhood Cancer Registry. Unfortunately, detailed data about treatments, location of the tumor, and recurrence is often incomplete or missing in the registry, so therefore we decided to use diagnosis (embryonal tumors, low grade astrocytomas, optic pathway gliomas, craniopharyngiomas, neuronal and mixed neuronal–glial tumors) to provide more specific information about different groups of the included cases. Coded data linked to each individual’s unique personal identification number were sent from the Swedish Childhood Cancer Registry to Statistics Sweden and matched to about five controls each by birth year, sex, and place of living at diagnosis. Each control was matched to only one PBTS and children treated for any types of cancer were not eligible as controls. Statistics Sweden provided information about whether the individuals studied Swedish as the first or second language during compulsory school and also about parental education, a potential confounding factor, as several studies have shown a strong correlation between parental education and student achievements [48,49]. All data were deidentified before analysis and only Statistics Sweden had access to the key code. Statistics Sweden also provided data about the subjects’ post-compulsory education, that is, attendance at the third (final) year in high school or at any time in municipal adult education, folk high school, and/or university from the years 2006–2017. Post-compulsory education variables only contain information about participation, not graduation.

### 2.2. Setting

Until 2018 the Swedish education system consisted of nine years of compulsory school. After compulsory school, there were three optional years, school years 10–12, herein referred to as “high school”. Our previous studies have shown that PBTS in Sweden are at risk of lower grades in both theoretical [23] and practical [46] school subjects, have lower scores on national tests [47], and are thus less qualified for high school than matched controls. These results are in line with other international studies on school performance [37,38,39]. There are various types of post-compulsory education in Sweden that teenagers and young adults can attend, some of them even without first obtaining a degree from compulsory school or high school. One option is to attend the municipal adult education (“Komvux” in Swedish) for those who need to retake courses that they failed during compulsory school or want to obtain a new education later in life. The municipal adult education also has courses or entire programs at the high school level. Moreover, for students who wish to have an accessible learning environment that is adapted to individual needs [43,44], there are the Swedish folk high schools (“folkhögskola” in Swedish). These schools often offer for example various types of esthetic and vocational programs for people with or without special needs. For higher education, Sweden has both universities and university colleges, with the same admission processes, herein collectively referred to as “university”. If a student has passed a theoretical program from high school, municipal adult education, or folk high school, or has taken extra courses in a vocational program to obtain university eligibility, they can apply to university educations in general, but some university programs have specific entry requirements. High school, municipal adult education, folk high school, and university are all free of charge, except for books and other study materials. All students may apply for study grants or study loans when studying at least part-time.

### 2.3. Statistical Analyses

Statistical analyses were performed using IBM SPSS Statistics for Windows, version 28.0.1.0 (IBM Corp., New York, NY, USA). *p*-values < 5% were considered statistically significant. Pearson’s chi-square test was used for comparison between PBTS’ and controls’ background variables. Differences between PBTS versus controls and females versus males are described with odds ratios (OR) and 95% confidence intervals (CI) and tested with logistic regressions in which attendance at high school, municipal adult education, folk high school, and university were used as dependent variables and being PBTS or control as independent variables. Two different interaction effects were tested in these logistic regressions to analyze whether the impact of sex (female or male) differed between PBTS and controls or if the impact of mothers’ and fathers’ education levels differed between PBTS or controls. Differences between the subgroups embryonal tumors, low-grade astrocytomas, optic pathway gliomas, craniopharyngiomas, neuronal and mixed neuronal–glial tumors and controls were also compared using logistic regressions and described as odds ratios (OR) and 95% confidence intervals (CI). Participation in high school, municipal adult education, folk high school, and university were used as dependent variables and tumor type and mothers’ education as independent variables. We chose to include mothers’ education as mothers’ education seem to be more important than fathers’ education for the child’s educational success [50,51]. Numbers equal to or below 5 in the analyses are only marked with <5 to ensure participant integrity, and due to low case numbers and these cases OR were not calculated.

## 3. Results

### 3.1. Background Factors

We found no significant differences between PBTS and controls regarding sex (*p* = 0.976), parental education (mothers’ education *p* = 0.345; fathers’ education *p* = 0.292) or whether participants studied Swedish as a first or second language (*p* = 0.653).

### 3.2. No Post-Compulsory Education

Controls were 2.06 times more likely than individuals in the PBTS group to attend any post-compulsory education (post-compulsory education includes high school, municipal adult education, folk high school, and university; Table 2). For both PBTS and controls, fewer males than females attended post-compulsory education (Table 2). Parental education had a significant impact on post-compulsory education for all study subjects, with low education levels associated with a higher risk of no post-compulsory education (Table 2). Age at diagnosis had not any apparent effect on the lack of post-compulsory education. When the subgroups embryonal tumors, low grade astrocytomas, optic pathway gliomas, craniopharyngiomas, neuronal and mixed neuronal–glial tumors were compared with controls, we found that the group treated for embryonal tumors and the group treated for optic pathway gliomas had less post-compulsory education compared with controls.

### 3.3. Post-Compulsory Education

We observed significant differences between PBTS and controls regarding the frequency of high school, university, and folk high school attendance, but no significant differences in municipal adult education attendance (Table 3). Significantly fewer PBTS compared with controls attended the third year of high school, and this was the same for both females and males (Table 3). Parental education affected high school attendance, with higher maternal or paternal education associated with higher post-compulsory education attendance for both PBTS and controls. We found no significant differences between PBTS diagnosed at different ages. When the subgroups of different tumor types were compared with controls, we found that the group treated for embryonal tumors, and the group treated for optic pathway gliomas attended the third year in high school less frequently than controls. Municipal adult education attendance did not differ between PBTS and controls (Table 3), but a larger proportion of females attended municipal adult education in both groups (PBTS and controls). Parental education did not have an impact on municipal adult education attendance for either PBTS or controls. There were no significant effects of age at diagnosis. When the subgroups of different tumor types were compared with controls, we found no significant differences for municipal adult education attendance. PBTS attended folk high schools more frequently than controls (Table 3). We observed no significant differences between females and males in folk high school attendance for either group (PBTS or controls). Neither did parental education, nor did age at diagnosis, affect folk high school attendance. When the subgroups of different tumor types were compared with controls, we found that the group treated for embryonal tumors, and the group treated for craniopharyngiomas attended folk high schools significantly more often than controls. Significantly fewer PBTS compared with controls attended university, and for both groups, more females than males attended university (Table 3). Parents’ education had a large impact on university attendance, both for PBTS and controls, with a positive correlation between the parents’ education levels and the study subjects’ university attendance. University attendance did not differ between PBTS diagnosed at different ages. When the subgroups of different tumor types were compared with controls, we found that the group treated for embryonal tumors, and the group treated for optic pathway gliomas, attended university less often than controls. Taken together, our analyses showed that females attended university and municipal adult education significantly more often than males in both PBTS and control groups. Parents’ education levels significantly impacted high school and university attendance in both PBTS and controls. Age at diagnosis did not have any significant impact for PBTS for any of the different types of post-compulsory educations. PBTS diagnosed with embryonal tumors and PBTS diagnosed with optic pathway gliomas attended third year in high school and university the least and PBTS treated for embryonal tumors and craniopharyngiomas attended folk high school significantly more often compared with controls.

## 4. Discussion

We explored post-compulsory education attendance and its effect on employment in Swedish teenagers and young adults treated for brain tumors in childhood and found that fewer PBTS compared with controls attended the third year in high school and university. This was expected, as our previous studies [23,46,47] among other studies [37,38,39] showed that children treated for brain tumors perform worse during their last year in compulsory school compared with their peers, and the present results confirm that these differences remain over time. When analyzing different tumor types separately, we found that PBTS treated for embryonal tumors and optic pathways gliomas attended the third year in high school and university significantly less than controls. This is not surprising as embryonal tumors, and their treatments, are particularly prone to giving rise to severe late complications that affect educational outcomes [20,21]. Moreover, children diagnosed with optic pathway gliomas may also, apart from the risk of visual impairment, have further complications due to a neurofibromatosis type 1-diagnosis [27,29], as mentioned in the introduction. There were trends towards low-grade astrocytomas, craniopharyngiomas and neuronal and mixed neuronal–glial tumors attending third year in high school and university less often than controls, but these differences were not significant. However, there was a significantly larger number of PBTS attending folk high schools compared with controls, especially PBTS treated for embryonal tumors and craniopharyngiomas. Folk high schools often have a more accessible and inclusive learning environment [43,44] and some folk high schools even offer specific programs for individuals with acquired brain injuries [52]. This suggests that attending folk high schools is a common choice for PBTS with late complications. Similar results from a study on Swedish children treated for lymphoma [53] support this hypothesis.

Younger age at diagnosis is a well-known risk factor for more severe late complications [4,6,12,13], but in this study the differences between the groups diagnosed at different ages were not significantly different for any of the assessed variables. This may be due to the low number of participants in each age group or, as discussed previously, other factors than neurocognitive deficits, such as visual impairment or other late complications that may affect everyday life. As expected, parental education had a large impact on high school and university attendance for all included participants. Subjects whose parents had a university education or higher were three times more likely to attend the third year in high school than those with parents without university education and subjects whose parents had a university education or higher were five times more likely to attend university than those with parents without university education. The impact of parental education did not differ between PBTS and controls. Parental education as a protective factor is in line with our previous studies and also a study by Ach et al. [54]. However, we could not find any evidence that parental education had an impact on folk high school or municipal adult education attendance. We can only speculate, but one reason could be that those who attended folk high schools did so for other reasons than academic ones, as discussed above. Similarly, municipal adult education is often more accessible than other options, may have fewer prerequisites, and students are able to take just one or two courses, for example if they had failed them earlier. In summary, parents’ education had a substantial effect only on higher education attendance, high school and university, but not on lower, more accessible, levels of education.

In summary, our results show that many individuals treated for a brain tumor in childhood are at risk for not obtaining any post-compulsory education or participate in less post-compulsory education than peers, especially children treated for embryonal tumors and optic pathways gliomas. As the risk of unemployment in Sweden is particularly high for young adults without post-compulsory education and for those with disabilities [45], it is of importance to provide PBTS with extra support also during the years of post-compulsory education, and to provide different types of post-compulsory education, for example folk high schools. As with healthy peers, parental education impacts educational attainment of PBTS and thus should be considered in support programs where extra attention should be given to PBTS without highly educated parents.

## 5. Strengths and Weaknesses

The major strength of this study is that it is a nationwide study where all children born between 1988 and 1996, diagnosed with a brain tumor before their fifteenth birthday, and contained in the Swedish Childhood Cancer Registry, were included. This registry comprises information about 93.2% of all children in Sweden treated for cancer [55] and good quality data on age at diagnosis and tumor type. We also had reliable national data from recent years about educational attainment and virtually all parents’ education from national registries at Statistics Sweden [56]. A limitation of the study is the lack of information for each individual about late effects such as visual impairment or hearing loss, different kind of treatments, location of the tumor, or recurrence rate. Another limitation is that we had no information about whether study participants passed their education only that they attended. In the future, when this cohort is large enough, it would be valuable to follow up this study cohort and look more closely at both highest exam and income.

## Figures and Tables

**Table 1:** Included pediatric brain tumor survivors (*n* = 452) and controls (n = 2188) and population characteristics describing age at diagnosis, tumor classification, Swedish as first or second language, and parents’ education.

	Pediatric Brain Tumor Survivors			Controls
	All *N* (%)	High-Grade * *N* (%) ^a^	Low-Grade * *N* (%) ^a^	All *N* (%)
All	452	80	372	2188
Females	218 (48.2)	37	181	1057 (48.3)
Males	234 (51.8)	43	191	1131 (51.7)
**Age at diagnosis**				
Females 0–5 years	80 (36.7)	20 (25.0) ^a^	60 (75.0) ^a^	
Females 6–9 years	48 (22.0)	10 (20.8) ^a^	28 (79.2) ^a^	
Females 10–14 years	90 (41.3)	7 (7.8) ^a^	83 (92.2) ^a^	
**Age at diagnosis**				
Males 0–5 years	83 (35.5)	14 (16.9) ^a^	69 (83.1) ^a^	
Males 6–9 years	63 (26.9)	8 (12.7) ^a^	55 (87.3) ^a^	
Males 10–14 years	88 (37.6)	21 (23.9) ^a^	67 (76.1) ^a^	
**Tumor classification**				
Ependymomas	27	9	18	
Choroid plexus tumors	10	1	9	
Astrocytomas	163	7	156	
Optic nerve gliomas	40	-	40	
Embryonal tumors (e.g., medulloblastoma and PNET ^b^)	52	52	-	
Oligodendrogliomas	11	1	10	
Mixed and unspecified gliomas	11		9	
Neuroepithelial glial tumors of uncertain origins	4	-	4	
Pituitary adenomas and carcinomas	10	2	8	
Craniopharyngiomas	31	-	31	
Pineal parenchymal tumors	8	3	5	
Neuronal and mixed neuronal–glial tumors	38	1	37	
Meningiomas	11	-	11	
Specified intracranial/intraspinal tumors	1	-	1	
Unspecified intracranial/intraspinal tumors	8	-	8	
Other specified/unspecified tumors	2	-	2	
Nerve sheath tumors	5	-	5	
Germ cell tumors	8	1	7	
Non-CNS ^c^ tumors by definition	12	-	12	
**Swedish as the first or second** **language**				
As the first language	429 (94.9)			2092 (95.6)
As the second language	23 (5.1)			96 (4.4)
**Mothers’ education**				
Low (school years 1–9 or less)	36 (8.0)			218 (10.0)
Medium (high school) ^d^	221 (48.9)			1083 (49.5)
High (higher education)	193 (42.7)			881 (40.3)
Missing information about education	2 (0.4)			6 (0.3)
**Fathers’ education**				
Low (school years 1–9 or less)	77 (17.0)			349 (16.0)
Medium (high school) ^d^	218 (48.2)			1151 (52.6)
High (higher education)	148 (32.7)			660 (30.2)
Missing information about education	9 (2.0)			28 (1.3)

* Tumor type. ^a^ This percentage indicates the difference between high-grade and low-grade tumors within the age groups. ^b^ Primitive neuro-ectodermal tumors. ^c^ Central nervous system. ^d^ Until 1994, high school could be two (mainly vocational programs) or three (theoretical programs) years in Sweden.

**Table 2:** No post-compulsory education any time between 2006–2017 for pediatric brain tumor survivors (PBTS; *n* = 452) and controls (*n* = 2188). Statistically significant results are marked with *.

No Post-Compulsory Education	*N* (%)	OR (95% CI; *p*)
PBTS vs. control group	37 (8.2) vs. 91 (4.2)	2.06 (1.38–3.05; <0.001) *
Sex (all boys vs. all girls)	81 (5.9) vs. 47 (3.7)	0.61 (0.42–0.88; 0.008) *
Interaction effect PBTS or control × sex		*p* = 0.100
Mothers’ education High vs. low High vs. medium Medium vs. low Missing information	29 (2.7) vs. 27 (10.6) 29 (2.7) vs. 72 (5.5) 72 (5.5) vs. 27 (10.6) *n* = 8	*p* < 0.001 * 0.23 (0.14–0.40; <0.001) * 0.48 (0.31–0.73; <0.001) * 0.49 (0.31–0.78; 0.003) *
Fathers’ education High vs. low High vs. medium Medium vs. low Missing information	18 (2.2) vs. 41 (9.6) 18 (2.2) vs. 66 (4.8) 66 (4.8) vs. 41 (9.6) *n* = 3	*p* < 0.001 * 0.21 (0.12–0.38; <0.001) * 0.45 (0.26–0.76; 0.003) * 0.48 (0.32–0.71; <0.001) *
Interaction effect PBTS or control × mothers’ education		*p* = 0.364
Interaction effect PBTS or control × fathers education		*p* = 0.061
Differences between age groups ^a^		*p* = 0.429
Embryonal tumors vs. all controls	9 (17.3) vs. 91 (4.2)	0.20 (0.09–0.42; <0.001) *
Low-grade astrocytomas vs. all controls	11 (7.1) vs. 91 (4.2)	0.57 (0.30–1.10; 0.095)
Optic pathway gliomas vs. all controls	7 (17.5) vs. 91 (4.2)	0.18 (0.08–0.43; <0.001) *
Craniopharyngiomas vs. all controls	<5 vs. 91 (4.2)	-
Neuronal and mixed neuronal–glial tumors vs. all controls	<5 vs. 91 (4.2)	-

^a^ Only PBTS included.

**Table 3:** Post-compulsory education any time between 2006–2017 for pediatric brain tumor survivors (PBTS) and controls. Statistically significant results are marked with *.

**Third Year in High School**	***N* (%)**	**OR (95% CI; *p*-Value)**
PBTS vs. control group	378 (83.6) vs. 1969 (90.0)	0.57 (0.43–0.76; <0.001) *
Sex (all boys vs. all girls)	1205 (88.3) vs. 1142 (89.6)	0.88 (0.69–1.12; 0.291)
Interaction effect PBTS or control × sex		*p* = 0.494
Mothers’ education High vs. low High vs. medium Medium vs. low Missing information	994 (92.6) vs. 199 (78.3) 994 (92.6) vs. 1147 (88.0) 1147 (88.0) vs. 199 (78.3) *n* = 7	*p* < 0.001 * 3.59 (2.46–5.24; <0.001) * 1.72 (1.29–2.28; <0.001) * 2.09 (1.48–2.94; <0.001) *
Fathers’ education High vs. low High vs. medium Medium vs. low Missing information	750 (92.8) vs. 345 (81.0) 750 (92.8) vs. 1220 (89.1) 1220 (89.1) vs. 345 (81.0) *n* = 32	*p* < 0.001 * 3.05 (2.13–4.38; 0.001) * 1.60 (1.17–2.21; 0.003) * 1.90 (1.41–2.56; <0.001) *
Interaction effect PBTS or control × mothers’ education		*p* = 0.473
Interaction effect PBTS or control × fathers’ education		*p* = 0.054
Differences between age groups ^a^		*p* = 0.685
Embryonal tumors vs. all controls	13 (25) vs. 1969 (90.0)	0.32 (0.17–0.62; <0.001) *
Low-grade astrocytomas vs. all controls	135 (86.5) vs. 1969 (90.0)	0.72 (0.44–1.16; 0.179)
Optic pathway gliomas vs. all controls	29 (72.5) vs. 1969 (90.0)	0.27 (0.13–0.55; <0.001) *
Craniopharyngiomas vs. all controls	27 (87.1) vs. 1969 (90.0)	0.58 (0.20–1.68; 0.311)
Neuronal and mixed neuronal–glial tumors vs. all controls	31 (81.6) vs. 1969 (90.0)	0.43 (0.19–1.00; 0.050)
**Municipal Adult Education**	***N* (%)**	**OR (95% CI; *p*)**
PBTS vs. control group	120 (26.6) vs. 602 (27.5)	0.95 (0.76–1.20; 0.676)
Sex (all boys vs. all girls)	311 (22.8) vs. 411 (32.2)	0.62 (0.52–0.74; <0.001) *
Interaction effect PBTS or control × sex		(*p* = 0.226)
Mothers’ education		*p* = 0.135
Fathers’ education		*p* = 0.312
Interaction effect PBTS or control ×mothers’ education		*p* = 0.716
Interaction effect PBTS or control × fathers education		*p* = 0.887
Differences between age groups ^a^		*p* = 0.643
Embryonal tumors vs. all controls	15 (28.8) vs. 602 (27.5)	1.07 (0.58–1.97; 0.823)
Low-grade astrocytomas vs. all controls	41 (26.3) vs. 602 (27.5)	0.94 (0.65–1.36; 0.736)
Optic pathway gliomas vs. all controls	9 (22.5) vs. 602 (27.5)	0.78 (0.37–1.64; 0.506)
Craniopharyngiomas vs. all controls	11 (35.5) vs. 602 (27.5)	1.53 (0.73–3.23; 0.259)
Neuronal and mixed neuronal–glial tumors vs. all controls	11 (28.9) vs. 602 (27.5)	1.10 (0.54–2.24; 0.787)
**Folk High School**	***N* (%)**	**OR (95% CI; *p*)**
PBTS vs. control group	69 (15.3) vs. 192 (8.8)	1.87 (1.39–2.52; <0.001) *
Sex (all boys vs. all girls)	124 (9.1) vs. 137 (10.7)	0.82 (0.64–1.07; 0.151)
Interaction effect PBTS or control × sex		*p* = 0.064
Mothers’ education		*p* = 0.312
Fathers’ education		*p* = 0.840
Interaction effect PBTS or control × mothers’ education		*p* = 0.536
Interaction effect PBTS or control × fathers education		*p* = 0.592
Differences between age groups ^a^		*p* = 0.084
Embryonal tumors vs. all controls	11 (21.2) vs. 192 (8.8)	2.78 (1.40–5.49; 0.003) *
Low-grade astrocytomas vs. all controls	18 (11.5) vs. 192 (8.8)	1.35 (0.81–2.26; 0.247)
Optic pathway gliomas vs. all controls	7 (17.5) vs. 192 (8.8)	2.18 (0.95–5.01; 0.065)
Craniopharyngiomas vs. all controls	8 (25.8) vs. 192 (8.8)	3.38 (1.49–7.69; 0.004) *
Neuronal and mixed neuronal–glial tumors vs. all controls	6 (15.8) vs. 192 (8.8)	1.89 (0.78–4.60; 0.156)
**University**	***N* (%)**	**OR (95% CI; *p*)**
PBTS vs. control group	165 (36.5) vs. 997 (45.6)	0.68 (0.55–0.84; <0.001) *
Sex (all boys vs. all girls)	512 (37.5) vs. 650 (51.0)	0.58 (0.49–0.67; <0.001) *
Interaction effect PBTS or control × sex		*p* = 0.525
Mothers’ education High vs. low High vs. medium Medium vs. low Missing information	637 (59.3) vs. 59 (23.2) 637 (59.3) vs. 463 (35.5) 463 (35.5) vs. 59 (23.2) *n* = 3	*p* < 0.001 * 5.29 (3.84–7.29; <0.001) * 2.75 (2.32–3.26; <0.001) * 1.49 (1.17–1.90; 0.001) *
Fathers’ education High vs. low High vs. medium Medium vs. low Missing information	534 (66.2) vs. 116 (27.2) 534 (66.2) vs. 497 (36.3) 497 (36.3) vs. 116 (27.2) *n* = 15	*p* < 0.001 * 5.40 (4.15–7.02; <0.001) * 3.62 (3.00–4.37; <0.001) * 1.92 (1.40–2.64; 0.001) *
Interaction effect PBTS or control × mothers’ education		*p* = 0.889
Interaction effect PBTS or control × fathers education		*p* = 0.710
Differences between age groups ^a^		*p* = 0.245
Embryonal tumors vs. all controls	12 (23.1) vs. 997 (45.6)	0.33 (0.17–0.64; 0.001) *
Low-grade astrocytomas vs. all controls	61 (39.1) vs. 997 (45.6)	0.76 (0.53–1.07; 0.110)
Optic pathway gliomas vs. all controls	12 (30) vs. 997 (45.6)	0.46 (0.23–0.94: 0.032) *
Craniopharyngiomas vs. all controls	11 (35.5) vs. 997 (45.6)	0.48 (0.22–1.02; 0.055)
Neuronal and mixed neuronal–glial tumors vs. all controls	15 (39.5) vs. 997 (45.6)	0.68 (0.35–1.34; 0.265)

^a^ Only PBTS included.

## Data Availability

Data may be obtained from a third party and are not publicly available. The data that support the findings of this study are available from the Swedish Childhood Cancer Registry and Statistics Sweden. Restrictions apply to the availability of these data, which were used under license for this study.
